# Blueprint for Building and Sustaining a Cardiogenic Shock Program: Qualitative Survey of 12 US Programs

**DOI:** 10.1016/j.jscai.2024.102288

**Published:** 2024-10-17

**Authors:** Raymond M. Yau, Robyn Mitchell, Aasim Afzal, Timothy J. George, Syed Siddiqullah, Aditya S. Bharadwaj, Alexander G. Truesdell, Carolyn Rosner, Mir B. Basir, Ruth Fisher, Allison Dupont, Carlos Leon Alviar, Haval Chweich, Navin K. Kapur, Rajan A.G. Patel, Scott Silvestry, Sandeep M. Patel, Jacob Abraham

**Affiliations:** a Heart Hospital of New Mexico, Albuquerque, New Mexico; b Heart Recovery Center, Baylor Scott & White The Heart Hospital − Plano, Plano, Texas; c Division of Cardiology, Loma Linda University, Loma Linda, California; d Virginia Heart, Falls Church, Virginia; e Inova Schar Heart and Vascular, Inova Fairfax Medical Campus, Falls Church, Virginia; f Division of Cardiovascular Diseases, Henry Ford Hospital, Detroit, Michigan; g Heart & Vascular Center, Moses Cone Hospital, Greensboro, North Carolina; h Northside Hospital Heart Institute, Atlanta, Georgia; i The Leon H. Charney Division of Cardiology, New York University Grossman School of Medicine & Bellevue Hospital, New York, NY; j Division of Pulmonary, Critical Care and Sleep Medicine, Tufts Medical Center and Tufts University School of Medicine, Boston, Massachusetts; k Department of Cardiology, The CardioVascular Center, Tufts Medical Center, Boston, Massachusetts; l John Ochsner Heart and Vascular Institute, Ochsner Medical Center, New Orleans, Louisiana; m Department of Surgery, College of Medicine, University of Arizona, Tucson, Arizona; n Mercy Health, St. Rita’s Medical Center, Lima, Ohio; o Center for Cardiovascular Analytics, Research + Data Science (CARDS), Providence Heart Institute, Providence Research Network, Portland, Oregon

**Keywords:** cardiogenic shock, protocol, shock teams

## Abstract

**Background:**

Multidisciplinary cardiogenic shock (CS) programs have been associated with improved outcomes, yet practical guidance for developing a CS program is lacking.

**Methods:**

A survey on CS program development and operational best practices was administered to 12 institutions in diverse sociogeographic regions and practice settings. Common steps in program development were identified.

**Results:**

Key steps for program development were identified: measuring baseline outcomes; identifying subspecialty champions; gaining leadership and team buy-in; developing institution-specific CS protocols; educating staff and referring providers; consulting with external experts; and developing quality assessment and process improvement.

**Conclusions:**

An assessment of 12 US CS programs highlights a blueprint for establishing and maintaining a successful, multidisciplinary shock program.

## Introduction

Cardiogenic shock (CS) is a heterogeneous, life-threatening syndrome. Despite improvements in the management of acute myocardial infarction (AMI), including regionalized AMI care and advances in percutaneous mechanical circulatory support (MCS) technology, in-hospital mortality for cardiogenic shock due to acute myocardial infarction (AMI-CS) remains persistently high.^1^, ^2^, ^3^, ^4^ Heart failure–related cardiogenic shock (HF-CS) has surpassed AMI-CS as the leading cause of CS and has an equally poor rate of 1-year survival, yet HF-CS remains poorly understood and its optimal treatment unclear.^5^^,^^6^ Regardless of the etiology of CS, delayed recognition of shock, late initiation of hemodynamic support, variations in critical care management, and inequities in access to CS centers contribute to poor patient outcomes.^7^, ^8^, ^9^, ^10^ Improving systems of care for cardiogenic shock (CS) is thus an urgent priority that transcends institutional boundaries.

Formalized CS programs have been developed to address these challenges, mirroring the approach used to combat other time-sensitive conditions requiring rapid, coordinated, and interdisciplinary care (eg, trauma, stroke, ST-segment elevation myocardial infarction). Indeed, in observational studies, implementation of a multidisciplinary CS team and a treatment protocol that prioritizes early right heart catheterization and use of MCS is associated with improved survival.^11^, ^12^, ^13^ The publication of the first positive randomized controlled trial of a MCS device in AMI-CS, coupled with the risks and costs of MCS, underscore the urgent need for health systems to implement best practices and institution-specific approaches to CS.^14^, ^15^, ^16^, ^17^, ^18^, ^19^

Although major cardiovascular societies have advocated development of CS programs, the challenges in starting and sustaining a CS program have been recently highlighted.^20^^,^^21^ These barriers to program success include ensuring adequate expertise in multiple clinical disciplines; training staff and maintaining competency; defining CS team oversight and leadership; maintaining team alignment; collecting data to ensure program quality; financing program costs; and ensuring sustainability at the individual, hospital, and system levels.^22^ There is a lack of practical guidance for navigating the formidable challenges to building and sustaining a CS program. We therefore undertook this descriptive study with the aim of providing a blueprint for implementing a CS program, drawing from the experience of clinical leaders at 12 high-volume institutions.

## Materials and methods

Representatives from a convenience sample of 12 institutions with CS programs located in diverse sociogeographical environments were invited to participate in a 1-hour interview with the lead author (R.Y.). Programs were considered eligible for participation in the survey if operational for at least 1 year, used a shock protocol, multidisciplinary shock team, and quality assurance. A questionnaire was designed after small group discussions (R.Y., J.A, A.T., and R.M) based on authors’ multiyear experiences in building their own shock teams and consulting with external institutions regarding development and maintenance of a CS program (Supplemental Material). We reviewed responses for commonly expressed practices in CS program development and operation. Common themes were those identified among a simple majority of respondents’ answers and subsequently expanded upon through group discussion. A final collection of best practices was agreed upon by consensus, including use of institutional data as an impetus for overcoming inertia; need for a multidisciplinary team approach; joint development of shock protocols; and continuing quality improvement review.

## Results

Characteristics of programs represented in this survey are summarized in Tables 1 and 2. Based on individual interviews conducted by the lead author and follow-on group discussions, 7 common steps were identified by consensus as best practices in the development of CS programs (Central Illustration).

**Table 1:** Characteristics of community shock program participants

	Mercy Health − St Rita's Medical Center	Heart Hospital of New Mexico	Northside Hospital	Providence St. Vincent Medical Center	Advent Health
Location	Lima, Ohio	Albuquerque, New Mexico	Atlanta, Georgia	Portland, Oregon	Orlando, Florida
Program start year	2021	2021	2021	2009	2008
No. of hospital beds/ICU beds	500/30	50/16	600/20	550/28	1200/32
LVAD program	No	Yes	No	Yes	Yes
Heart transplant program	No	No	No	Yes	Yes
Employment model	Employed	Employed	Mixed	Employed	Employed
MCS devices	IABP pLVAD pRVAD TS-LVAD Transeptal-pLVAD ECMO	IABP pLVAD pRVAD TS-LVAD Transeptal-pLVAD ECMO	IABP pLVAD pRVAD TS-LVAD ECMO	IABP pLVAD pRVAD TS-LVAD ECMO	IABP pLVAD pRVAD TS-LVAD Transeptal-pLVAD ECMO

IABP, intra-aortic balloon pump; ICU, intensive care unit; ECMO, extracorporeal membrane oxygenation; MCS, mechanical circulatory support; pLVAD, percutaneous left ventricular assist device; pRVAD, percutaneous right ventricular assist device; TS-LVAD, temporary surgical left ventricular assist device.

**Table 2:** Characteristics of academic shock program participants

	Henry Ford Hospital	Tufts Medical Center	New York University Langone Medical Center	Baylor Scott & White The Heart Hospital	Ochsner Medical Center	Inova Fairfax Medical Center	Loma Linda University Medical Center
Location	Detroit, Michigan	Boston, Massachusetts	New York, New York	Plano, Texas	New Orleans, Louisiana	Fairfax, Virginia	Loma Linda, California
Program start year	2008	2016	2011	2021	2022	2016	2020
No. of hospital beds/ICU beds	1000/150	350/28	957/26	107/60	1000/100	923/102	600/24
LVAD program	Yes	Yes	Yes	Yes	Yes	Yes	Yes
Transplant program	Yes	Yes	Yes	No	Yes	Yes	Yes
Employment model	Employed	Employed	Employed	Employed	Employed	Mixed	Employed
MCS devices	IABP pLVAD pRVAD TS-LVAD Transeptal-pLVAD ECMO	IABP pLVAD pRVAD TS-LVAD Transeptal-pLVAD ECMO	IABP pLVAD pRVAD TS-LVAD ECMO	IABP pLVAD pRVAD TS-LVAD ECMO	IABP pLVAD pRVAD TS-LVAD Transeptal-pLVAD ECMO	IABP pLVAD pRVAD TS-LVAD ECMO	IABP pLVAD pRVAD TS-LVAD ECMO

IABP, intra-aortic balloon pump; ICU, intensive care unit; ECMO, extracorporeal membrane oxygenation; MCS, mechanical circulatory support; pLVAD, percutaneous left ventricular assist device; pRVAD, percutaneous right ventricular assist device; TS-LVAD, temporary surgical left ventricular assist device.

**Central Illustration:**
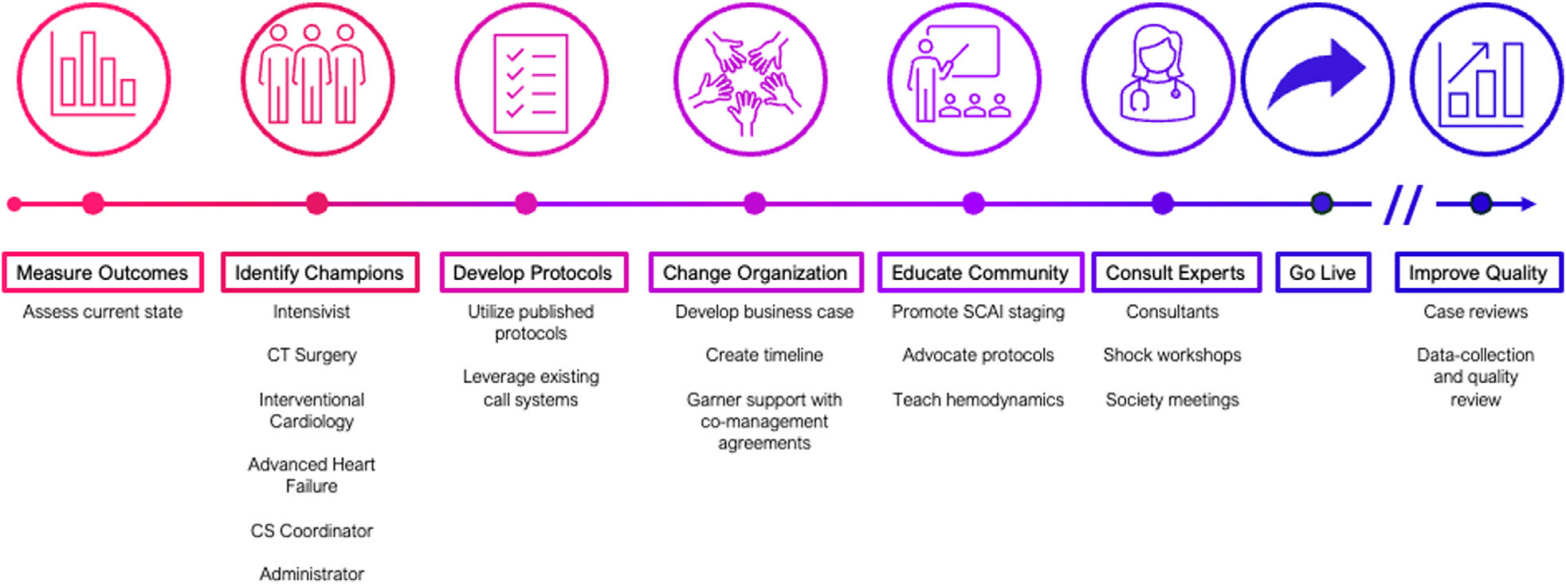
**Blueprint for CS program development.** CS, cardiogenic shock; CT, computed tomography.

## Discussion

### Step 1: Measure baseline outcomes

The initial step in developing a CS program is collecting data to characterize the local CS population, identify gaps in care and establish baseline outcomes. Collection of center-specific CS data will highlight opportunities for improvement. Retrospective analysis using electronic medical record (EMR) reports related to CS diagnosis codes was most practical for many institutions.

Collection of center-specific or system-specific CS data can highlight opportunities for care improvement. While there are no guideline-endorsed quality benchmarks specific to CS shock management, comparison of center data with those reported in contemporary, multicenter observational registries (Cardiogenic Shock Working Group, American Heart Association Shock Registry, Extracorporeal Life Support Registry) allows for benchmarking.

### Step 2: Identify champions

In this process of needs assessment and data collection, clinician champions, ideally representing all relevant medical specialties, are instrumental for leading discussions and driving progress with other clinicians and administrators. A clinician champion should consider a stakeholder analysis to identify peers and senior leadership with whom to form a coalition for organizational change.

### Step 3: Organizational change

Organizational change requires buy-in from all stakeholders including administrators, clinical providers, ancillary service lines, and varies depending on practice setting. It is often the most difficult step in starting a CS program but can be overcome.

A sustainable CS program requires institutional support for personnel, training, and capital equipment. Garnering support from administrators should focus on advocating for the clinical need for a CS program while presenting a compelling business case. Key components of the business case should include an analysis of case volumes, impact on contribution margin, and benefits to other clinical programs. A proposal that outlines implementation timelines, expected outcomes, and measurable success indicators will highlight feasibility. A typical timeline for CS program development is 6 to 12 months.

In a nonemployed/private practice model, barriers to developing a CS program may include misalignment of incentives and competing interests. Aligning team members with different compensation models and incentive structures is challenging. It is important for physician and administrative leadership to engage with all stakeholders so that areas of mutual risk and reward can be addressed. Incentives for participation may be considered at institutions with multiple medical groups. Leadership roles could be offered to individuals representing different practices, ensuring that all provider groups feel invested.

Traditional academic medical centers may offer distinct challenges and opportunities to CS program startup. Academic medical centers often have greater financial constraints or competing priorities, making it difficult to secure resources, and often also have established advanced heart failure (AHF) and cardiothoracic surgery divisions whose members will play essential roles in a CS program.

Regardless of practice setting, a comanagement agreement may be an effective tool to ensure alignment between clinicians and administrators. This legal contract between the hospital administration and physician group incentivizes performance that improves outcomes, efficiency, or revenue. For example, the cardiology group at Loma Linda University Medical Center included performance metrics of frequency of CS team activation and adherence to shock protocol in a comanagement agreement. This initiative resulted in a significant increase in their cardiologists activating shock teams and adhering to their institutions’ shock protocol.

Once a CS program is established, it is important to celebrate the “early wins.” Sharing patient stories builds enthusiasm within the institution and local community. A regular cadence of communication and progress reports with the leadership team maintains transparency and accountability.

### Step 4: CS team and protocol development

Composition of the CS team will be specific to each institution. At small community hospitals, interventional cardiologists and intensivists typically comprise CS teams. MCS devices are implanted and managed by these subspecialists, respectively. At larger centers, CS teams typically include AHF and cardiothoracic surgery and may include a dedicated CS nurse coordinator, critical care nursing, perfusion, and/or respiratory therapy.

A single call process, ideally one that uses existing processes for ST-elevation MI or stroke, enables efficient communication and rapid mobilization of personnel.^12^ An advantage of leveraging an existing call system is that clinicians are already familiar with the process. The CS team should have 24/7 availability; at smaller hospitals, smartphone applications or EMR-based chat applications may facilitate communication. Our collective experience highlights the importance of including at least 1 physician champion in the shock team discussion, at least in the early stages, to avoid reverting to a status quo approach. Additionally, experience suggests that even high-performing institutions can improve by implementation of a structured, team-based approach. Several of the programs interviewed have adopted publicly available protocols.^4^^,^^13^ The widespread adoption of Society for Cardiovascular Angiography & Interventions (SCAI) stages of CS can be useful in protocol design to match treatment of severity of shock.^14^^,^^15^ Protocols should be formulated with clear inclusion/exclusion criteria and binary decision trees (Supplemental Material). HF-CS has eclipsed AMI-CS as the predominant etiology.^16^ Patients with HF-CS more often present with congestion, are more likely to undergo heart replacement therapies (left ventricular assist device, heart transplant), and have lower in-hospital mortality.^17^, ^18^, ^19^ Use of protocols with etiology-specific treatment pathways is associated with improved survival and is endorsed by society guidelines.^21^

It is important for an emerging CS program to integrate into existing networks of CS care and identify the appropriate level of care it should provide. Smaller centers should partner with centers capable of providing advanced support (ie, high-flow MCS devices, extracorporeal membrane oxygenation, biventricular support) and left ventricular assist device or heart transplant. Recent statements from the International Society for Heart and Lung Transplantation on HF-CS underscore the need for creating such networks of care to ensure appropriate identification and timing of transfer to an AHF center.^22^^,^^23^

### Step 5: Team and referring education

Shock protocols should be simple, agreed upon, and widely disseminated. Widescale education can be achieved by posting the care protocol in high-traffic areas such as cardiac catheterization laboratory, telemetry floors, and intensive care units (ICUs) or by distributing pocket cards to key providers. Educational sessions on cardiovascular hemodynamics, MCS insertion and management, and protocol navigation can aid acceptance and adoption of the CS protocol in early stages. Maintaining educational competencies can be challenging at new sites, especially smaller sites where volumes can be low. This dilemma further reinforces the need for not just physician champions but also champions in other allied fields such as nursing and perfusion, to concentrate adequate experience to gain expertise. Advanced practice providers may allow for around-the-clock on-site cardiac ICU staffing with dedicated midlevel experts to bridge potential care gaps created by nursing and physician staffing changes. Off-site electronic ICU monitoring facilities may similarly provide more cost-effective and resource-friendly solutions to smaller facilities.

Establishing effective educational initiatives within the hub shock facility and collaborating with other health systems can contribute to the development of a larger CS network of care. Priority should be given to disseminating information regarding CS recognition, SCAI staging, CS algorithms, transfer protocols, and logistics. Respondents cited dinners, workshops, and conferences as successful educational venues. Case review can also be used as an educational opportunity for internal and external referring teams and allows for continuous quality improvement.

### Step 6: Expert consultation

Creating a CS program requires effort and commitment from multiple stakeholders. Consulting with medical experts in the field can accelerate the startup process. For example, Heart Hospital of New Mexico hosted experts from Inova Heart. This well-attended event increased buy-in from skeptical stakeholders and provided reassurance that all critical components were in place before launching the program. Similarly, Loma Linda University offers a formal educational program on shock teams. The agenda includes establishing a monthly shock meeting, creating order sets, obtaining buy-in from administrators, and other implementation tips.

Cardiovascular society sponsored conferences such as SCAI Shock and International Society for Heart and Lung Transplantation also offer practical guidance for emerging CS programs and propose solutions to common challenges, including administrative buy-in, from the perspective of both clinicians and administrators.

### Step 7: Quality metrics and continuing review

Robust collection and monitoring of quality measures is paramount to continuous quality improvement. Monitoring adherence to institutional protocols, survival to discharge, adverse event rates, transfer volumes, and other metrics may help identify clinical trends and can help ensure appropriate resource allocation (Table 3).

**Table 3:** Suggested core metrics for quality assurance and program improvement.

Clinical metrics	Baseline and maximal SCAI-CSWG stage tMCS utilization Survival to discharge 30-day survival Vascular complications Renal-replacement therapy
Process metrics	Shock team activation times Transfer volumes Time to transfer Time to MCS Adherence to protocol PAC utilization
System metrics	Length of stay 30-day readmission Staff training and certification

PAC, pulmonary artery catheter; SCAI-CSWG, Cardiogenic Shock Working Group interpretation of Society for Cardiovascular Angiography & Intervention; MCS, mechanical circulatory support.

Data collection among the 12 programs included in this survey varied significantly based on available resources, ranging from in-house databases to nationwide registries such as the Cardiogenic Shock Working Group Registry, AHA (American Heart Association) Get with the Guidelines Shock Registry, and the ELSO (Extracorporeal Life Support Organization) Registry. These larger databases make it efficient for programs to benchmark against similar programs and perform data query. Future EMR innovations and interoperability may simplify and advance these data collection.

A dedicated CS coordinator involved in program quality was a common thread among the CS programs included in this report. The job description of the CS coordinator often includes data collection, tracking of quality metrics, and implementation of quality measures.

The increasing prevalence of CS and rising utilization of MCS underscore the need for health systems to develop, maintain, and monitor systems of care for patients with this complex, highly lethal condition. Over the past decade, improved survival in both AMI-CS and HF-CS has been associated with implementation of CS teams equipped with institution-specific protocols of care.^11^^,^^13^ Translating these learnings into practical guidance is urgently need to close gaps in CS care delivery, particularly in underserved areas. In this qualitative, survey-based study involving representatives from 12 health systems in the United States, we sought to provide practical guidance to clinicians and administrators on creation of a CS program.

### Limitations

This report is a narrative-based, qualitative survey from a convenience sample of physicians from 12 US institutions with established CS programs. The survey was not developed nor validated using formal survey methodologies. Selection bias is introduced by surveying centers with established shock teams, protocols, and quality assurance programs. The collective responses are based on consensus expert opinion and must be adapted to local culture, community practice standards, and system resources.

## Conclusions

The development of a formal CS program is recommended to improve clinical outcomes. We conducted interviews with representatives from 12 high-volume CS programs across the United States to derive best practice recommendations for emerging CS programs. We identified common themes and summarized recommendations in a pragmatic narrative blueprint.

Despite different patient populations, institutional resources, and geographic locations, each of the programs represented has implemented a high-volume, high-quality CS program. Domains of consensus centered on the need for site-specific data on CS outcomes and process metrics; multidisciplinary engagement; development and implementation of a CS protocol; and need for quality assurance program.
