# Supplementary material for: Blueprint for Building and Sustaining a Cardiogenic Shock Program: Qualitative Survey of 12 US Programs
Source: J Soc Cardiovasc Angiogr Interv. 2024 Oct 17;3(11):102288. doi: 10.1016/j.jscai.2024.102288 (PMC11624379; doi:10.1016/j.jscai.2024.102288)
Supplement: Supplemental material [file mmc1.pdf]

Supplement
Cardiogenic Shock Protocols Used

Henry Ford Hospital and Northside Hospital

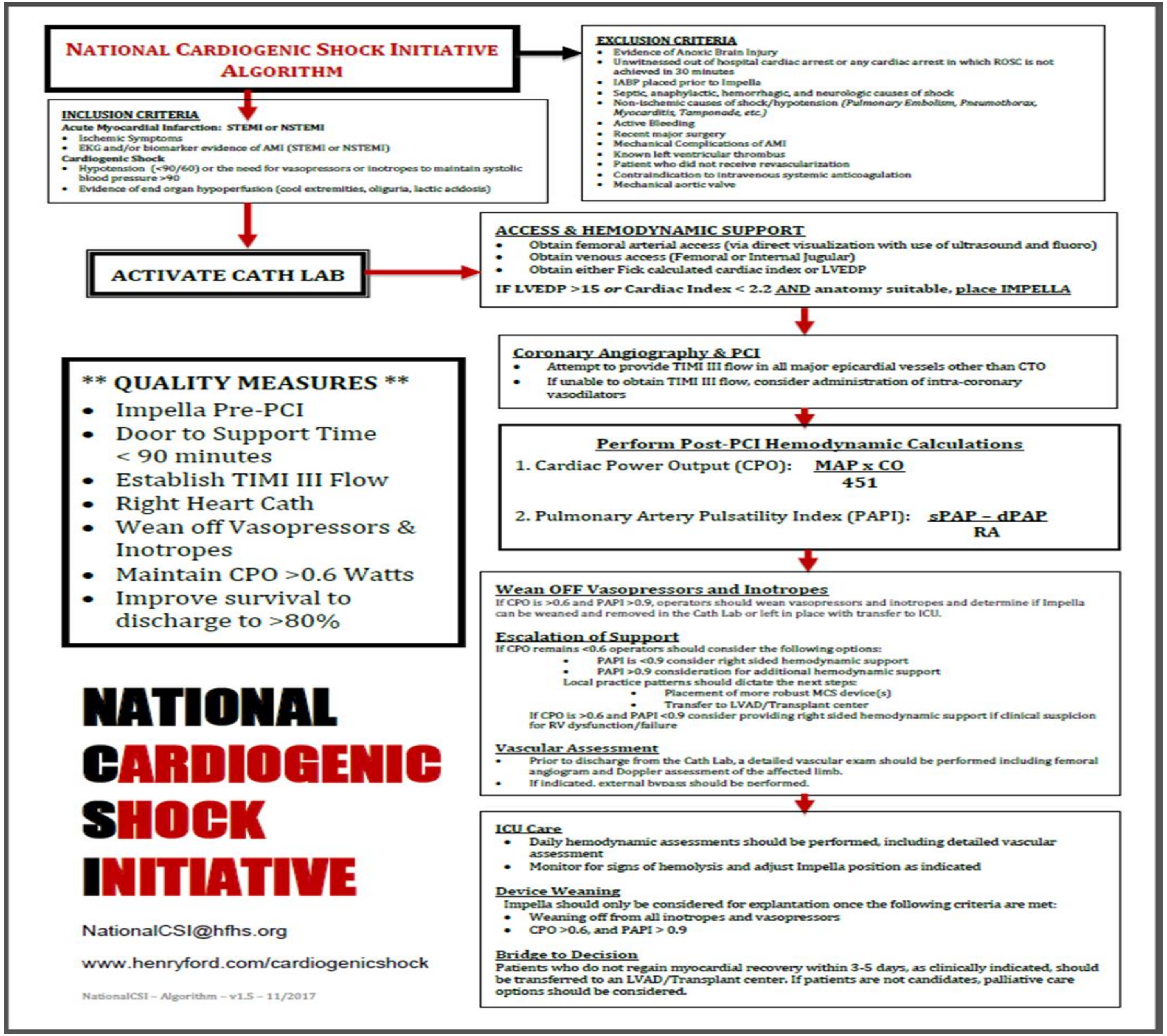

NATIONAL CARDIOGENIC SHOCK INITIATIVE ALGORITHM

INCLUSION CRITERIA

Acute Myocardial Infarction: STEMI or NSTEMI

- Ischemic Symptoms
- EKG and/or biomarker evidence of AMI (STEMI or NSTEMI)

Cardiogenic Shock

- Hypotension (<90/60) or the need for vasopressors or inotropes to maintain systolic blood pressure >90
- Evidence of end organ hypoperfusion (cool extremities, oliguria, lactic acidosis)

EXCLUSION CRITERIA

- Evidence of Anoxic Brain Injury
- Unwitnessed out of hospital cardiac arrest or any cardiac arrest in which ROSC is not achieved in 30 minutes
- IABP placed prior to Impella
- Septic, anaphylactic, hemorrhagic, and neurologic causes of shock
- Non-ischemic causes of shock/hypotension *(Pulmonary Embolism, Pneumothorax, Myocarditis, Tamponade, etc.)*
- Active Bleeding
- Recent major surgery
- Mechanical Complications of AMI
- Known left ventricular thrombus
- Patient who did not receive revascularization
- Contraindication to intravenous systemic anticoagulation
- Mechanical aortic valve

ACTIVATE CATH LAB

ACCESS & HEMODYNAMIC SUPPORT

- Obtain femoral arterial access (via direct visualization with use of ultrasound and fluoro)
- Obtain venous access (Femoral or Internal Jugular)
- Obtain either Fick calculated cardiac index or LVEDP

IF LVEDP >15 *or* Cardiac Index < 2.2 AND anatomy suitable, place IMPELLA

Coronary Angiography & PCI

- Attempt to provide TIMI III flow in all major epicardial vessels other than CTO
- If unable to obtain TIMI III flow, consider administration of intra-coronary vasodilators

Perform Post-PCI Hemodynamic Calculations

1. Cardiac Power Output (CPO): $\frac{MAP \times CO}{451}$
2. Pulmonary Artery Pulsatility Index (PAPI): $\frac{sPAP - dPAP}{RA}$

Wean OFF Vasopressors and Inotropes

If CPO is >0.6 and PAPI >0.9, operators should wean vasopressors and inotropes and determine if Impella can be weaned and removed in the Cath Lab or left in place with transfer to ICU.

Escalation of Support

If CPO remains <0.6 operators should consider the following options:

- PAPI is <0.9 consider right sided hemodynamic support
- PAPI >0.9 consideration for additional hemodynamic support

Local practice patterns should dictate the next steps:

- Placement of more robust MCS device(s)
- Transfer to LVAD/Transplant center

If CPO is >0.6 and PAPI <0.9 consider providing right sided hemodynamic support if clinical suspicion for RV dysfunction/failure

Vascular Assessment

- Prior to discharge from the Cath Lab, a detailed vascular exam should be performed including femoral angiogram and Doppler assessment of the affected limb.
- If indicated, external bypass should be performed.

ICU Care

- Daily hemodynamic assessments should be performed, including detailed vascular assessment
- Monitor for signs of hemolysis and adjust Impella position as indicated

Device Weaning

Impella should only be considered for explantation once the following criteria are met:

- Weaning off from all inotropes and vasopressors
- CPO >0.6, and PAPI > 0.9

Bridge to Decision

Patients who do not regain myocardial recovery within 3-5 days, as clinically indicated, should be transferred to an LVAD/Transplant center. If patients are not candidates, palliative care options should be considered.

**** QUALITY MEASURES ****

- Impella Pre-PCI
- Door to Support Time < 90 minutes
- Establish TIMI III Flow
- Right Heart Cath
- Wean off Vasopressors & Inotropes
- Maintain CPO >0.6 Watts
- Improve survival to discharge to >80%

NATIONAL CARDIOGENIC SHOCK INITIATIVE

NationalCSI@hfhs.org

www.henryford.com/cardiogenicshock

NationalCSI – Algorithm – v1.5 – 11/2017

Providence St. Vincent Medical Center

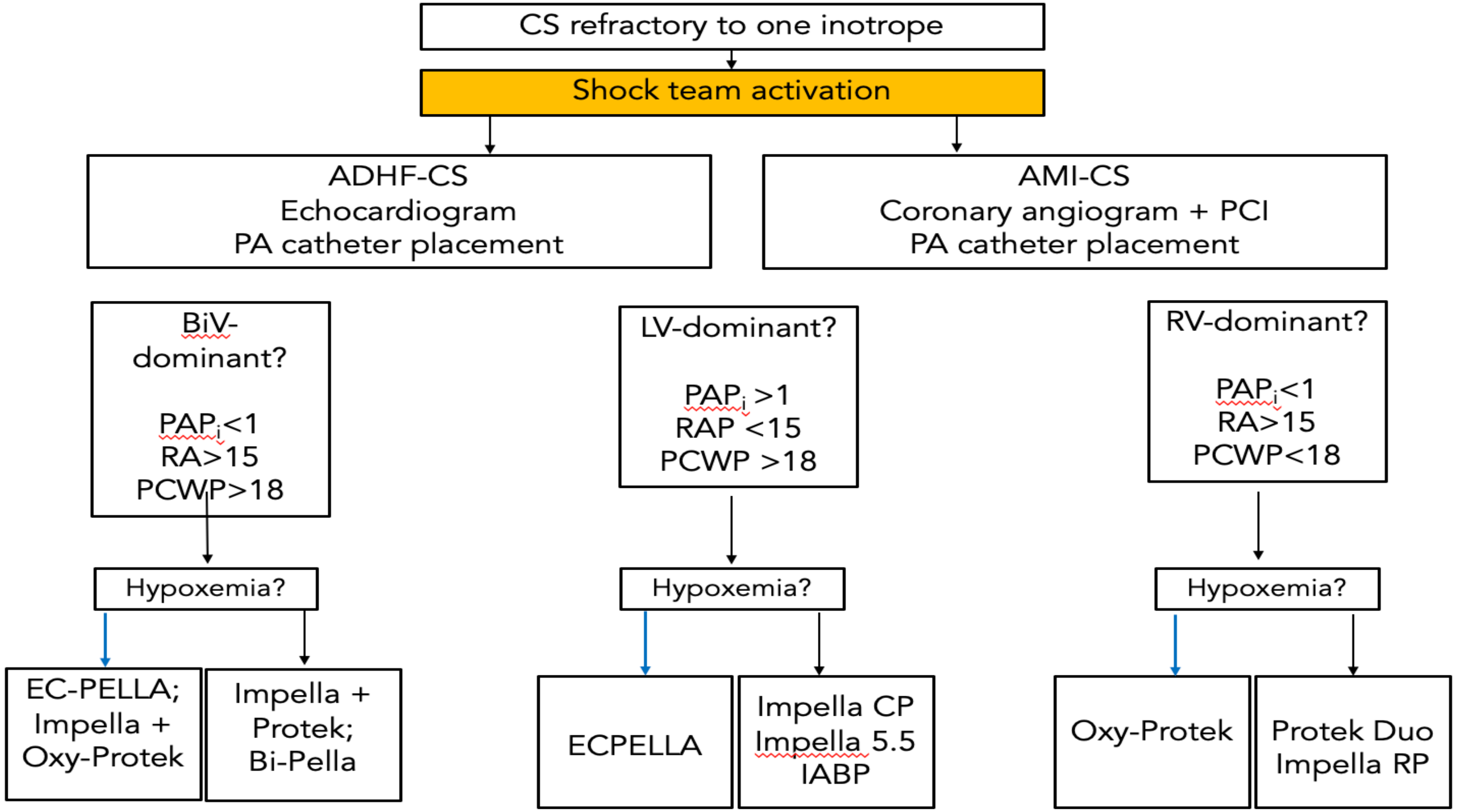

Inova Fairfax Medical Center

https://www.frontiersin.org/articles/10.3389/fcvm.2024.1354158/full

Inova — Cardiogenic Shock Team Activation → 703-776-8000

WHY is there a Shock Team?

Early identification and treatment improves survival in Cardiogenic Shock

WHAT is the Cardiogenic Shock Team?

A **multidisciplinary team** dedicated to optimizing the care of Cardiogenic Shock patients via:

- Rapid identification
- Coordinated consultation
- Early transfer/admission to Cardiac ICU, Cath Lab or Operating Room

WHO is on the Shock Team?

- Interventional Cardiologist
- Cardiac Surgeon
- Advanced Heart Failure
- Cardiac Critical Care

HOW is the Shock Team activated?

Inova Transfer Center: **703-776-5905**

WHO activates the Shock Team?

- Emergency Department
- Other units in the hospital (eg, Cath Lab or ICUs)
- Other hospitals

Revised May 3, 2022

WHEN is the Team Activated?

Call the Shock Team as soon as Cardiogenic Shock is suspected

Clinical Criteria

- SBP < 90mmHg (for 30 min) or use of vasopressors/inotropes
- Lactate > 2 mmol/L
- Evidence of end-organ (eg, renal, hepatic, cerebral) hypoperfusion
- ACS or Heart Failure

Hemodynamic Criteria (if known)

- CI < 1.8 (or 2.2 L/min/m² with inotropes or vasopressors)
- CPO < 0.6
- PAPi < 1.0
- PCWP ≥ 15 mmHg

Contraindications*

- DNAR
- Terminal Illness

❯❯ Note: for STEMI, follow STEMI pathway

If any questions, contact Shock Team

AFTER the team has been activated

- Obtain ongoing Vital Signs, ECG, Labs (eg, BNP, Tn I, Lactate, CBC, CMP)
- Maintain 2 large bore IVs (consider central line as needed)
- Minimize vasopressors/inotropes to maintain MAP of ≥ 60 mmHg
- Preferential use of norepinephrine for vasopressor support
- ***Avoid*** use of phenylephrine
- Preferential use of amiodarone for control of VT or AF
- ***Avoid*** negative inotropes (eg, β-blockers, Ca^{++} channel blockers)
- Consider airway stabilization

Inova — Cardiogenic Shock Team Coordination

Heart Team Goals

- Early identification of CS patients
- Early CS phenotyping
- Selective and tailored PMCS
- Optimize hemodynamics
- Native heart recovery

*Clinical Considerations for PMCS

- Shock phenotype (AMI-CS vs HF-CS)
- Shock severity (SCAI Classification)
- Shock profile (LV, RV, Bi-V)
- Lactate level
- Severity of end-organ dysfunction
- Amount of vasopressor/inotropic support
- Presence of hypoxia
- Presence of arrhythmias

Relative PMCS Contraindications

- DNAR
- Terminal illness
- Unable to anticoagulate
- Cardiac arrest with neurocatastrophe
- Advanced multi-system organ failure
- LA or LV thrombus

CPO = MAP x CO/451
PAPi = (sPAP-dPAP)/RA

Revised April 24, 2022

Cardiogenic Shock Team Activation

- Call **703-776-8000** for any patient with criteria for **Cardiogenic Shock**
- Obtain ongoing Vital Signs, ECG, Labs

HF-CS

- Echocardiography
- Right Heart Catheterization

AMI-CS

- Coronary angiography with LVEDP
- Right Heart Catheterization

Are Criteria for Cardiogenic Shock Met?

- **SBP < 90mmHg or use of vasopressors/inotropes AND:**
- **CI < 1.8 (or < 2.2 L/min/m² with inotropes/vasopressors)**
- **PCWP ≥ 15 mmHg and/or LVEDP ≥ 15 mmHg**
- **CPO < 0.6**
- **PAPi < 1.0**
- **Lactate > 2 mmol/L**
- **Evidence of end-organ hypoperfusion**

YES

- **Consider Percutaneous Mechanical Circulatory Support (PMCS) based on Clinical Considerations for PMCS***
- Coronary revascularization prn (consider IV antiplatelet agent)

NO

- Coronary revascularization as needed
- Swan-Ganz Catheter left in place

Cardiac Intensive Care Unit for ongoing CS Management

- **Serial reassessment of hemodynamics & end-organ perfusion**
- Optimize Preload, Afterload, and Contractility
- Timely, tailored *escalation of treatment* for **Worsening Shock**
- Assess for ability to wean PMCS

Inova — HF-Cardiogenic Shock Management

Call 703-776-8000 to activate Cardiogenic Shock Team

CS Management Goals

- ***Serial reassessment (≤ q 6hr)*** of hemodynamics & end-organ perfusion
  - Lactate
  - Renal, hepatic function
  - Continuous hemodynamics
  - CPO & PAPi
- Optimize *Preload, Afterload* and *Contractility*
  - Volume or diuresis
  - Vasodilators or Vasopressors
  - Inotropes
- Timely, tailored treatment *escalation* for **Worsening Shock:**
  - Rising Lactate
  - Increasing pressor requirement
  - Worsening end-organ function
  - CPO < 0.6 and/or PAPi < 1
  - RA > 15 and/or PCWP > 15
- Assess for LV and RV recovery
  - Wean PMCS, vasopressors and inotropes

CS Hemodynamic Profile

	LV-dominant	RV-dominant	Bi-V
**RA**	< 15	> 15	> 15
**PCWP**	> 15	< 15	> 15
**CPO**	< 0.6	< 0.6	< 0.6
**PAPi**	> 1.0	< 1.0	< 1.0

CPO = MAP x CO/451
PAPi = (sPAP-dPAP)/RA

Treatment Considerations for *Heart Failure-CS*

- ***Shock severity (SCAI stage)***
- ***Shock profile (LV, RV or Bi-V)***
- Anticipated exit strategy (BTT or BTR)
- Presence of hypoxia
- Presence of arrhythmias
- Anticipated duration of support
- Ability to ambulate
- Contraindications to PMCS

SCAI B CS — *Beginning*
Hypoperfusion: Lactate < 2 mmol/L; Minor renal & hepatic dysfunction
+/-
Hypotension: SBP < 90 mmHg
Current Treatment: No drugs or devices
→ **LV, RV or Bi-V:** IABP (and/or trial of vasopressors, inotropes or vasodilators)

SCAI C CS — *Classic*
Hypoperfusion: Lactate ≥ 2 mmol/L; Major renal & hepatic dysfunction
+
Hypotension: SBP < 90 mmHg
OR
Current Treatment: 1 drug OR device
→ **LV-dominant:** IABP or Impella 5.5
→ **RV-dominant or Bi-V:** Pro-Tek Duo +/- Impella 5.5

SCAI D CS — *Deteriorating*
Hypoperfusion: Lactate ≥ 4 mmol/L; Worsening renal & hepatic function
+
Hypotension: Escalating pressors
OR
Current Treatment: 2 drugs OR devices
→ **LV-dominant:** Impella 5.5 or Trans-apical or Trans-septal temporary LVAD
→ **RV-dominant or Bi-V:** VA-ECMO +/- LV vent

SCAI E CS — *Extremis*
Hypoperfusion: Lactate ≥ 8 mmol/L; Severe acidosis & end-organ failure
+
Hypotension: Refractory
OR
Current Treatment: ≥3 drugs OR devices
→ **LV, RV or Bi-V:** VA-ECMO +/- LV vent

Revised May 3, 2022

Inova — AMI-Cardiogenic Shock Management

Call 703-776-8000 to activate Cardiogenic Shock Team

CS Management Goals

- ***Serial reassessment (≤ q 6hr)*** of hemodynamics & end-organ perfusion
  - Lactate
  - Renal, hepatic function
  - Continuous hemodynamics
  - CPO & PAPi
- Optimize *Preload, Afterload* and *Contractility*
  - Volume or diuresis
  - Vasodilators or Vasopressors
  - Inotropes
- Timely, tailored treatment *escalation* for **Worsening Shock:**
  - Rising Lactate
  - Increasing pressor requirement
  - Worsening end-organ function
  - CPO < 0.6 and/or PAPi < 1
  - RA > 15 and/or PCWP > 15
- Assess for LV and RV recovery
  - Wean PMCS, vasopressors and inotropes

CS Hemodynamic Profile

	LV-dominant	RV-dominant	Bi-V
**RA**	< 15	> 15	> 15
**PCWP**	> 15	< 15	> 15
**CPO**	< 0.6	< 0.6	< 0.6
**PAPi**	> 1.0	< 1.0	< 1.0

CPO = MAP x CO/451

Treatment Considerations for *AMI-CS*

- ***Shock severity (SCAI stage)***
- ***Shock profile (LV, RV or Bi-V)***
- Revascularization status (mode and completeness)
- Presence of mechanical complications (eg, VSD, MR)
- Presence of hypoxia
- Presence of arrhythmias
- Contraindications to PMCS
- Use of IV antiplatelet agent

SCAI B CS — *Beginning*
Hypoperfusion: Lactate < 2 mmol/L; Minor renal & hepatic dysfunction
+/-
Hypotension: SBP < 90 mmHg
Current Treatment: No drugs or devices
→ **LV, RV or Bi-V:** IABP (and/or trial of vasopressors)

SCAI C CS — *Classic*
Hypoperfusion: Lactate ≥ 2 mmol/L; Alteration of renal & hepatic function
+
Hypotension: SBP < 90 mmHg
OR
Current Treatment: 1 drug OR device
→ **LV-dominant:** IABP or Impella CP
→ **RV-dominant or Bi-V:** Pro-Tek Duo +/- Impella CP

SCAI D CS — *Deteriorating*
Hypoperfusion: Lactate ≥ 4 mmol/L; Worsening renal & hepatic function
+
Hypotension: Escalating pressors
OR
Current Treatment: 2 drugs OR devices
→ **LV-dominant:** Impella 5.5 or VA-ECMO +/- LV vent
→ **RV-dominant or Bi-V:** VA-ECMO +/- LV vent

SCAI E CS — *Extremis*
Hypoperfusion: Lactate ≥ 8 mmol/L; Severe acidosis & end-organ failure
+
Hypotension: Refractory
OR
Current Treatment: ≥ 3 drugs OR devices
→ **LV, RV or Bi-V:** VA-ECMO +/- LV vent

New York University Langone Medical Center

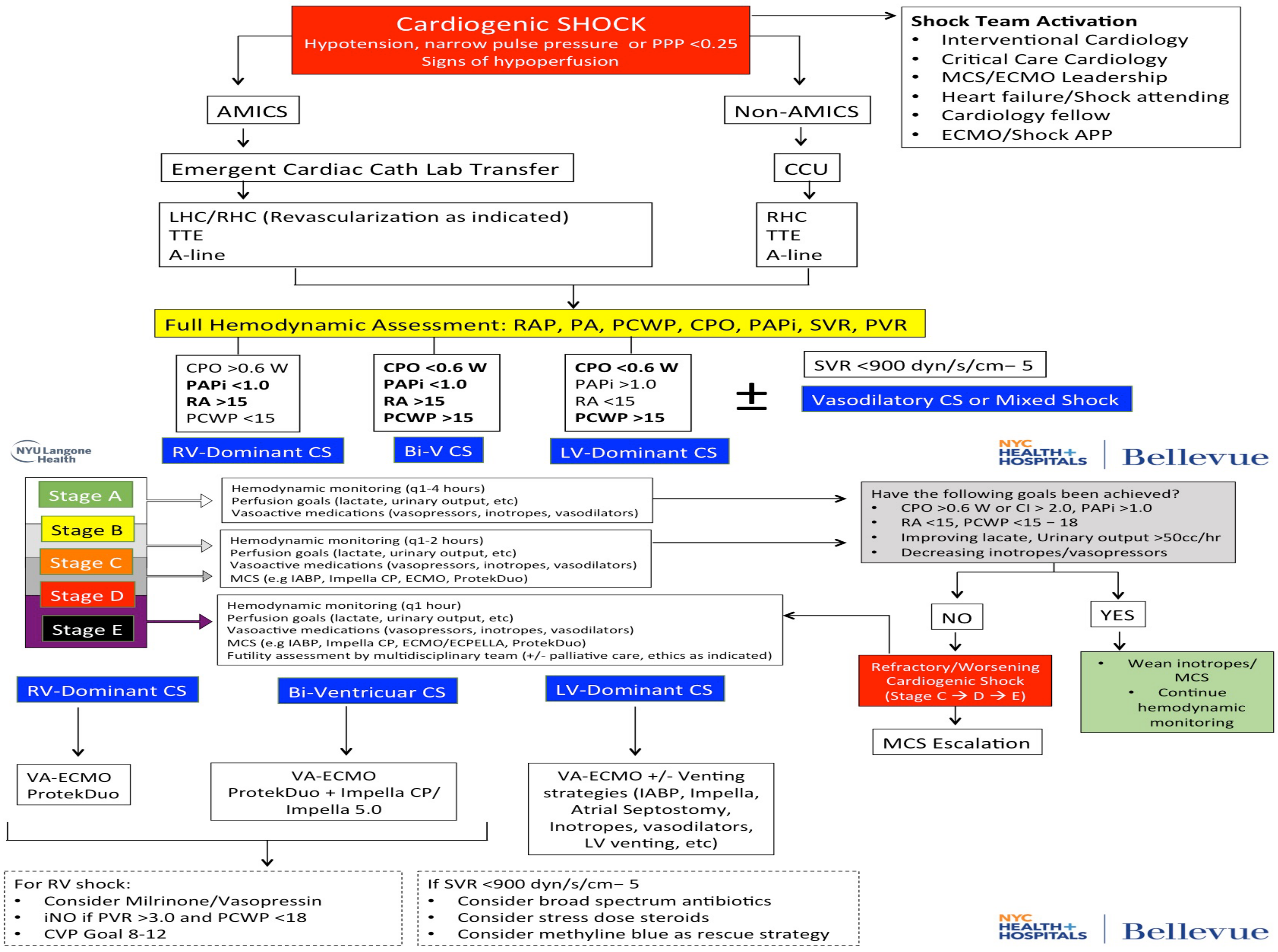

Mercy Health St Rita Medical Center

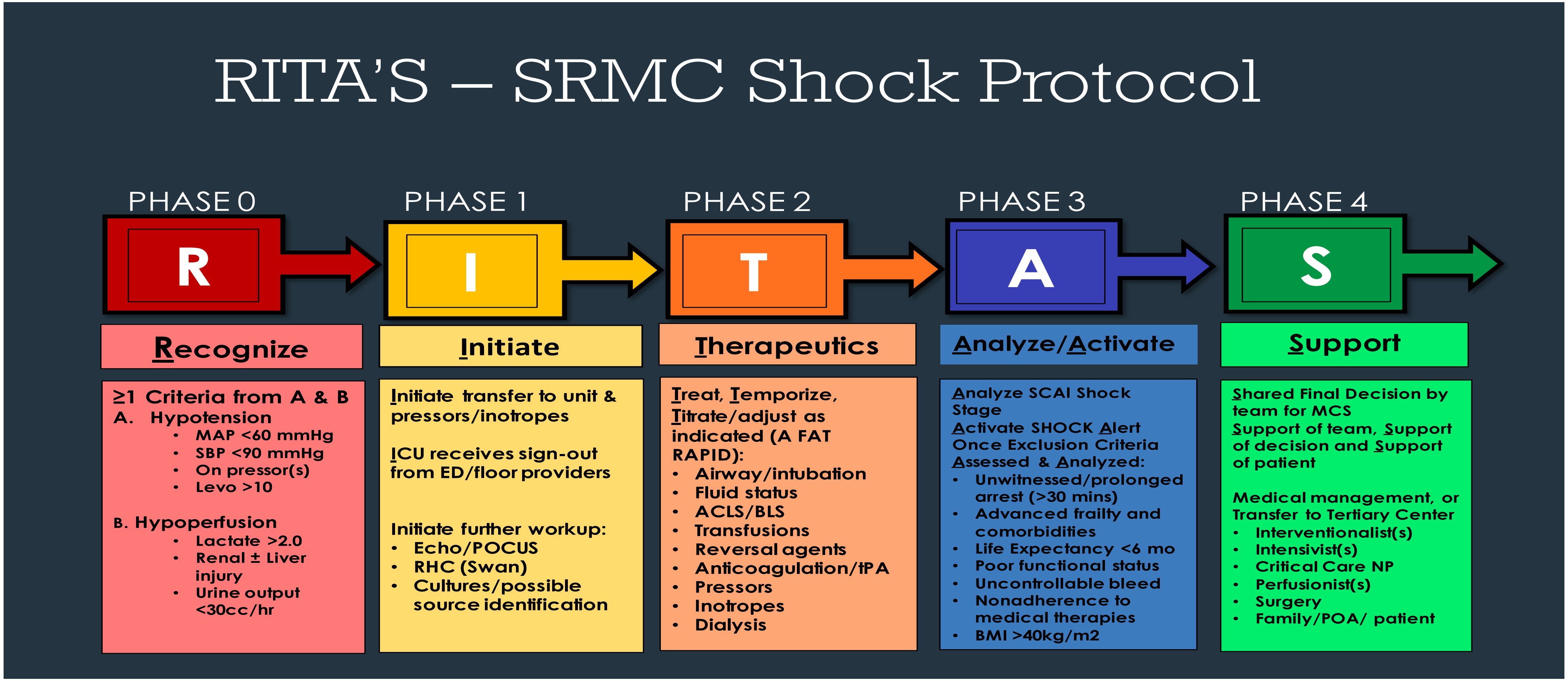

Tufts Medical Center

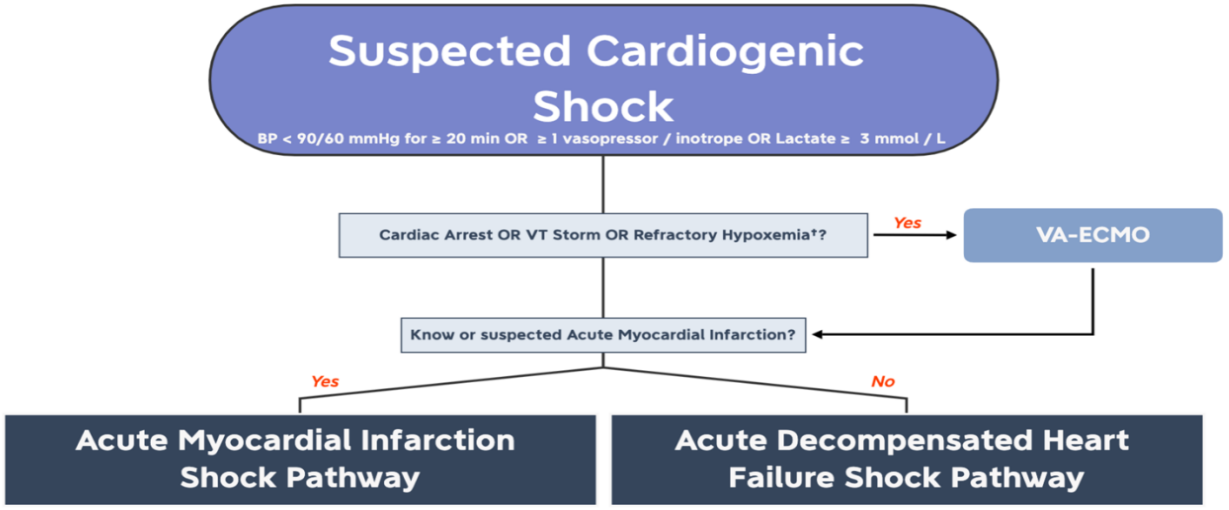

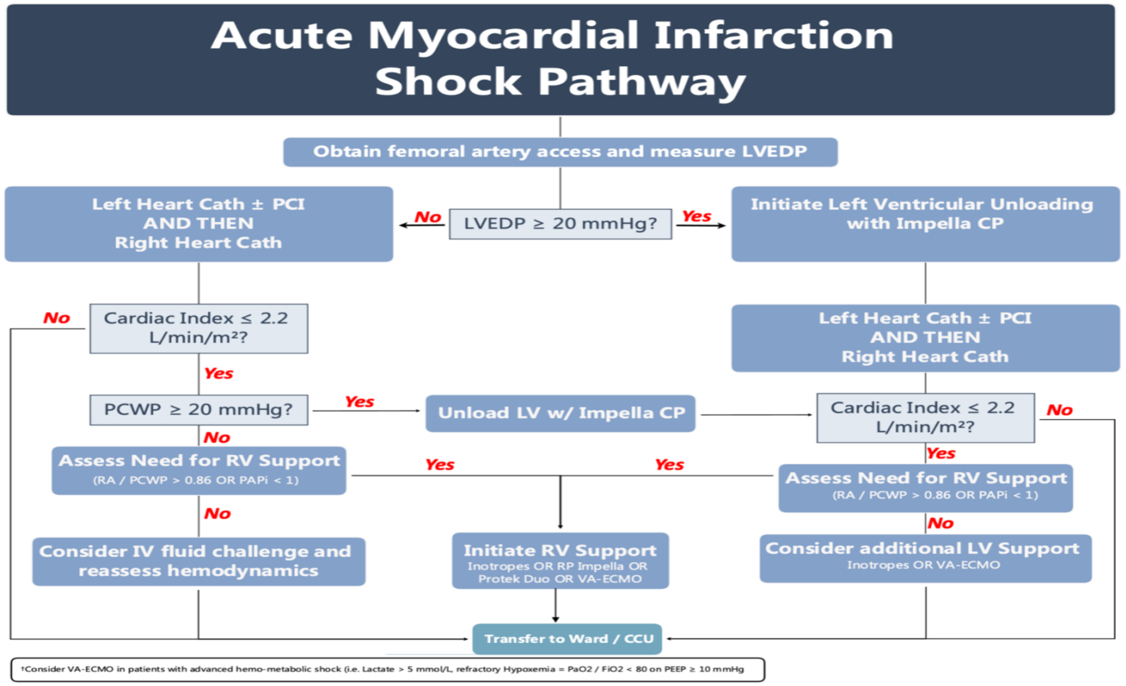

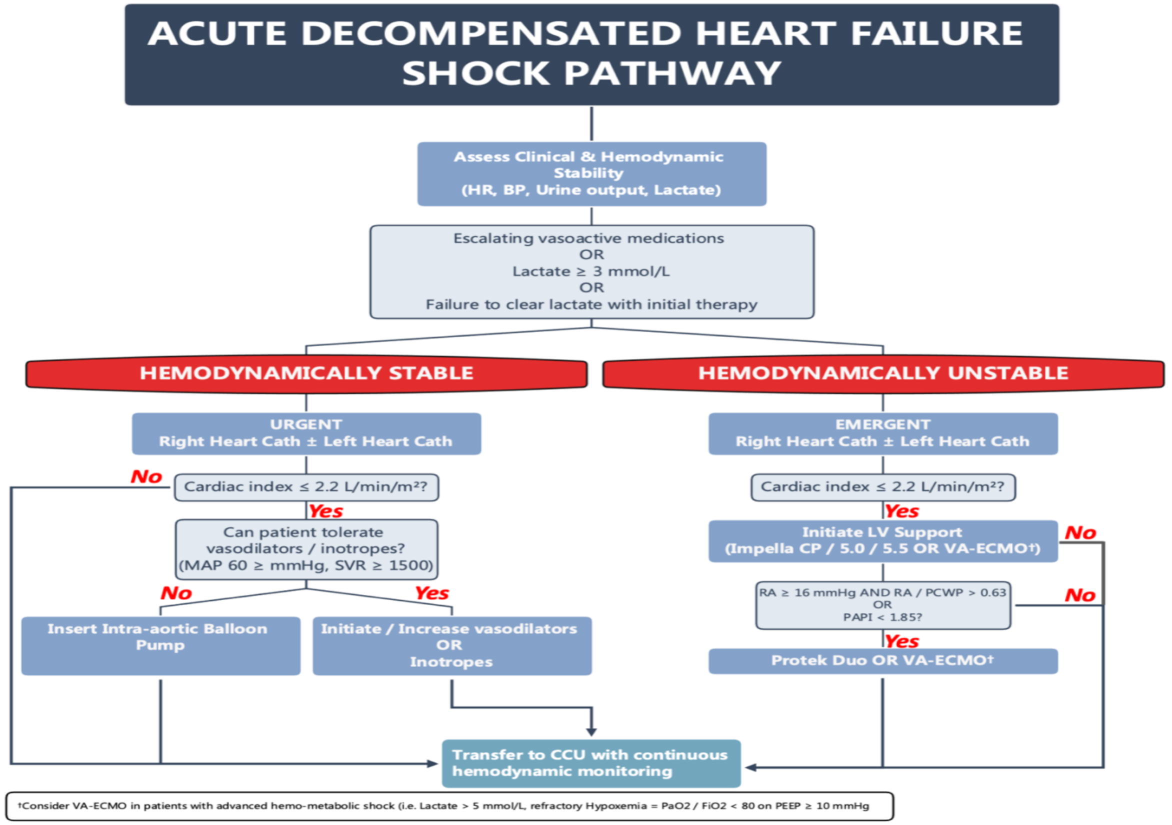

Advent Health

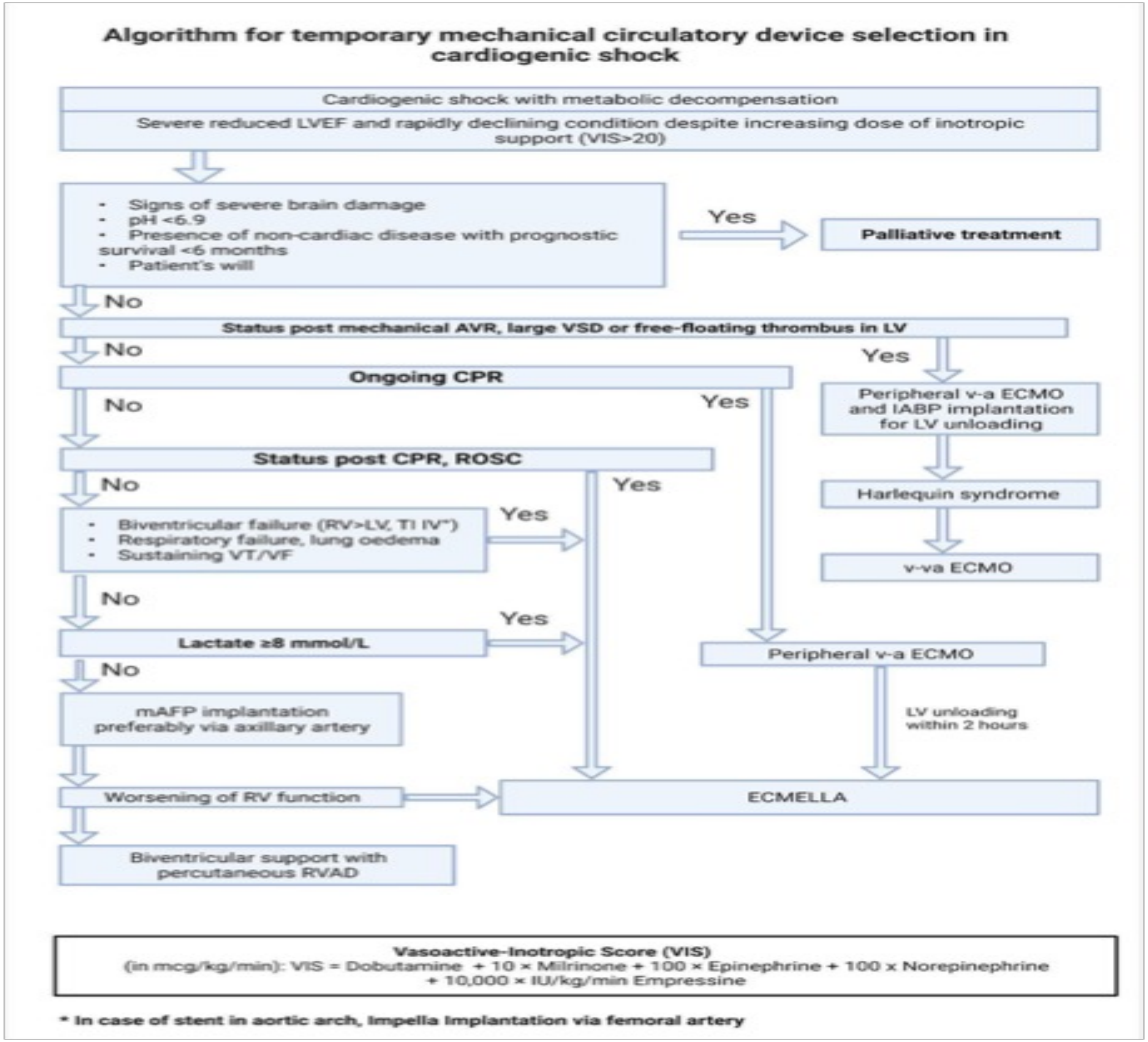

Heart Hospital of New Mexico

Cardiogenic Shock Team ACTIVATION

Call **888.687.6428** or **505.727.7646**

Heart Hospital of New Mexico @ LOVELACE MEDICAL CENTER — 100 YEARS

WHY is there a Shock Team?

Early identification and treatment improves survival in Cardiogenic Shock.

WHAT is the Cardiogenic Shock Team?

A **multidisciplinary team** dedicated to optimizing the care of Cardiogenic Shock patients via:
- Rapid identification
- Coordinated consultation
- Early transfer/admission to Cardiac ICU, Cath Lab or Operating Room

WHO is on the Shock Team?

- Interventional Cardiologist
- Cardiac Critical Care

HOW is the Shock Team activated?

Call **888.687.6428** or **505.727.7646**

WHO activates the Shock Team?

- Emergency Department
- Other units in the hospital (eg, Cath Lab or ICUs)
- Other hospitals

WHEN is the Team Activated?

Call the Shock Team as soon as Cardiogenic Shock is suspected

Clinical Criteria

Clinical presentation consistent acute decompensated heart failure or acute coronary syndrome with either:
- Hypotension. SBP<90 (for 30 min) or use of vasopressors/inotropes or
- Hypoperfusion Lactate>2, evidence of end-organ (eg, renal hepatic, cerebral) hypoperfusion

Hemodynamic Criteria (if known)
- CI < 1.8 (or 2.2 L/min/m² with inotropes or vasopressors)
- CPO < 0.6
- PAPi < 1.0
- PCWP ≥ 15 mmHg

*Contraindications**
- DNAR
- Terminal Illness

>> Note: for STEMI, follow STEMI pathway

*if any questions, contact the Cardiogenic Shock Team

AFTER the team has been activated

- Obtain ongoing vital signs, ECGs, Labs (eg, BNP, Tn I, Lactate, CBC, CMP)
- Maintain 2 large bore IVs (consider central line as needed)
- Minimize vasopressors/inotropes to maintain MAP of > 60 mmHG
- Preferential use of norepinephrine for vasopressor support
- Preferential use of amiodarone for control of VT or AF
- Avoid negative inotropes (eg, ß-blockers, Ca⁺⁺ channel blockers)
- Consider airway stabilization

Cardiogenic Shock Team COORDINATION

Heart Hospital of New Mexico @ LOVELACE MEDICAL CENTER — 100 YEARS

Heart Team Goals

- Early identification of CS patients
- Early CS phenotyping
- Selective and tailored PMCS
- Optimize hemodynamics
- Native heart recovery

* Clinical Considerations for PMCS

- Shock phenotype (AMI-CS vs HF-CS)
- Shock severity (SCAI Classification)
- Shock profile (LV, RV, Bi-V)
- Lactate level
- Severity of end organ dysfunction
- Amount of vasopressor/inotropic support
- Presence of hypoxia
- Presence of arrhythmias

Relative PCMS Contraindications

- DNAR
- Terminal illness
- Unable to anticoagulate
- Unable to receive blood products
- Cardiac arrest with neurocatastrophe
- Advanced multi-system organ failure
- LA or LV thrombus

*CPO=MAP x CO/451 *PAPi=(sPAP-dPAP)/RA

Heart Team Activation

- Call 888.687.6428 or 505.727.7646 for any patient with criteria for **Cardiogenic Shock**
- Obtain ongoing Vital Signs, ECG, Labs

HF-CS
- Echocardiography
- Right Heart Catheterization

AMI-CS
- Coronary angiography with LVEDP
- Right Heart Catheterization

Are Criteria for Cardiogenic Shock Met?

- **SBP < 90mmHg or use of vasopressors/inotropes AND/OR:**
- **CI < 1.8 (or < 2.2 L/min/m² with inotropes/ vasopressors)**
- **PCWP ≥ 15 mmHg and/or LVEDP ≥ 15 mmHg**
- **CPO < 0.6**
- **PAPi < 1.0**
- **Lactate > 2 mmol/L**
- **Evidence of end-organ hypoperfusion**

YES
- **Consider Percutaneous Mechanical Circulatory Support (PMCS) based on Clinical Considerations for PMCS***
- Coronary revascularization PRN (consider IV antiplatelet agent)

NO
- Coronary revascularization as needed
- Swan-Ganz catheter left in place

Cardiac Intensive Care Unit for ongoing CS Management

- **Serial reassessment of hemodynamics & end-organ perfusion**
- Optimize Preload, Afterload, and Contractility
- Timely, tailored escalation of treatment for **Worsening Shock**
- Assess for ability to wean PMCS

HF-Cardiogenic Shock MANAGEMENT

Call **888.687.6428** or **505.727.7646** to activate Heart Team

Heart Hospital of New Mexico @ LOVELACE MEDICAL CENTER — 100 YEARS

CS Management Goals

- ***Serial reassessment (≤ q 6hr)*** of hemodynamics & end-organ perfusion
  - Lactate
  - Renal, hepatic function
  - Continuous hemodynamics
  - CPO & PAPi
- Optimize *Preload, Afterload and Contractility*
  - Volume or diuresis
  - Vasodilators or Vasopressors
  - Inotropes
- Timely, tailored treatment escalation for **Worsening Shock:**
  - Rising Lactate
  - Increasing pressor requirement
  - Worsening end organ function
  - CPO < 0.6 and/or PAPi < 1
  - RA > 15 and/or PCWP > 15
- Assess for LV and RV recovery
  - Wean PMCS, vasopressors and inotropes

CS Hemodynamic Profile

	LV-dominant	RV-dominant	Bi-V
RA	< 15	>15	>15
PCWP	>15	< 15	>15
CPO	< 0.6	< 0.6	< 0.6
PAPi	> 1.0	< 1.0	< 1.0

CPO=MAP x CO/451 *PAPi=(sPAP-dPAP)/RA

Treatment Considerations for Heart Failure-CS

- **Shock severity (SCAI stage)**
- **Shock profile (LV, RV or Bi-V)**
- Anticipated exit strategy (BTT or BTR)
- Presence of hypoxia
- Presence of arrhythmias
- Anticipated duration of support
- Ability to ambulate
- Contraindications to PMCS

SCAI B CS — *Beginning*
Hypoperfusion: Lactate < 2 mmol/L; Minor renal & hepatic dysfunction
+/-
Hypotension: SBP < 90 mmHg
Current Treatment: No drugs or devices
→ **LV, RV or Bi V:** IABP (and/or trial of vasopressors, inotropes or vasodilators)

SCAI C CS — *Classic*
Hypoperfusion: Lactate ≥ 2 mmol/L; Major renal & hepatic dysfunction
+
Hypotension: SBP < 90 mmHg
OR
Current Treatment: 1 drugs OR device
→ **LV-dominant:** IABP or Impella CP or Trans-septal temporary LVAD
→ **RV-dominant or Bi-V:** Pro-Tek Duo +/- Impella CP or Trans-septal temporary LVAD

SCAI D CS — *Deteriorating*
Hypoperfusion: Lactate ≥ 4 mmol/L; Worsening renal & hepatic dysfunction
+
Hypotension: Escalating pressors
OR
Current Treatment: 2 drugs OR devices
→ **LV-dominant:** Impella 5.5 or Trans-apical or temporary LVAD
→ **RV-dominant or Bi-V:** VA-ECMO +/- LV vent

SCAI E CS — *Extremis*
Hypoperfusion: Lactate ≥ 8 mmol/L; Severe acidosis & end-organ failure
+
Hypotension: Refractory
OR
Current Treatment: ≥ 3 drugs OR devices
→ **LV, RV or Bi-V:** VA-ECMO +/- LV vent

AMI-Cardiogenic Shock MANAGEMENT

Call **888.687.6428** or **505.727.7646** to activate Heart Team

Heart Hospital of New Mexico @ LOVELACE MEDICAL CENTER — 100 YEARS

CS Management Goals

- ***Serial reassessment (≤ q 6hr)*** of hemodynamics & end-organ perfusion
  - Lactate
  - Renal, hepatic function
  - Continuous hemodynamics
  - CPO & PAPi
- Optimize *Preload, Afterload and Contractility*
  - Volume or diuresis
  - Vasodilators or Vasopressors
  - Inotropes
- Timely, tailored treatment escalation for **Worsening Shock:**
  - Rising Lactate
  - Increasing pressor requirement
  - Worsening end organ function
  - CPO < 0.6 and/or PAPi < 1
  - RA > 15 and/or PCWP > 15
- Assess for LV and RV recovery
  - Wean PMCS, vasopressors and inotropes

CS Hemodynamic Profile

	LV-dominant	RV-dominant	Bi-V
RA	< 15	>15	>15
PCWP	>15	< 15	>15
CPO	< 0.6	< 0.6	< 0.6
PAPi	> 1.0	< 1.0	< 1.0

CPO=MAP x CO/451 *PAPi=(sPAP-dPAP)/RA

Treatment Considerations for AMI-CS

- **Shock severity (SCAI stage)**
- **Shock profile (LV, RV or Bi-V)**
- Revascularization status (mode and completeness)
- Presence of mechanical complications (eg, VSD, MR)
- Presence of hypoxia
- Presence of arrhythmias
- Contraindications to PMCS
- Use of IV antiplatelet agent

SCAI B CS — *Beginning*
Hypoperfusion: Lactate < 2 mmol/L; Minor renal & hepatic dysfunction
+/-
Hypotension: SBP < 90 mmHg
Current Treatment: No drugs or devices
→ **LV, RV or Bi V:** Impella CP

SCAI C CS — *Classic*
Hypoperfusion: Lactate ≥ 2 mmol/L; Alteration of renal & hepatic dysfunction
+
Hypotension: SBP < 90 mmHg
OR
Current Treatment: 1 drugs OR device
→ **LV-dominant:** Impella CP or Transeptal temporary LVAD
→ **RV-dominant or Bi-V:** Pro-Tek Duo +/- Impella CP or Transeptal temporary LVAD

SCAI D CS — *Deteriorating*
Hypoperfusion: Lactate ≥ 4 mmol/L; Worsening renal & hepatic dysfunction
+
Hypotension: Escalating pressors
OR
Current Treatment: 2 drugs OR devices
→ **LV-dominant:** Impella 5.5 or VA-ECMO or Transeptal temporary LVAD
→ **RV-dominant or Bi-V:** VA-ECMO +/- LV vent

SCAI E CS — *Extremis*
Hypoperfusion: Lactate ≥ 8 mmol/L; Severe acidosis & end-organ failure
+
Hypotension: Refractory
OR
Current Treatment: ≥ 3 drugs OR devices
→ **LV, RV or Bi-V:** VA-ECMO +/- LV vent

Adopted by INOVA Heart and Vascular Institute

Ochsner Medical Center

OMC Cardiogenic Shock Team Algorithm

CONTRAINDICATIONS to MCS
- DNR
- Terminal illness
- Anoxic brain injury
- Unable to anticoagulated
- Advanced multi-system organ failure
- CAHP score > 200

INCLUSION CRITERIA: Cardiogenic Shock (HF or ACS)
- SBP < 90 for > 30 min
- Pressor/inotrope to keep SBP > 90
- CI < 1.8 without or < 2.2 with pressor/inotrope
- Evidence of end organ hypoperfusion
  - UO < 50cc/hr
  - Lactate > 2
  - Cool extremities
  - LFT's
- STEMI or NSTEMI
  - Clinical ischemia
  - Biomarker +
  - ECG +

Activate SHOCK TEAM

Begin treatment for ACS or HF (consider IABP)
AND Assess Criteria for Shock:
- Inotrope support- epi, nitric, nitroprusside, diuresis
- IJ Access, Art line,
- RHC, ECHO
- CVP, CO/CI, SVR; hypo-perfusion labs

RV Dysfunction Criteria
- PAPi < 1
- RA > 15 mmHg
- TAPSE < 14 to 17

HEMODYNAMIC CRITERIA
- Fick CI < 1.8L/min/m2 w p/i
- Fick CI < 2.2 L/min/m2 wo p/i
- PCWP > 18 mm Hg
- CPO < 0.7
- PAPi < 1.0

CPO = (MAPxCO)/451
PAPi = (sPAP–dPAP)/RA

Refractory Shock with initial MCS Platform

Y

LV	RV	Bi-V
CPO < .7	CPO < .7	CPO < .7
PAPi > 1.0	PAPi < 1.0	PAPi < 1.0
RA < 15	RA > 15	RA > 15
PCW > 15	PCW < 15	PCW > 15
Hypoxemia ?	Hypoxemia ?	Hypoxemia ?
Y: TH + O_2 or VA ECPELLA	Y: ProDu + O_2 or VA EC +/- PELLA	Y: ProDu +O_2 or VA ECPELLA
N: Impella CP or Impella 5.5	N: Impella RP Or ProDu	N: Bipella or TH/ProDu

N

CPO > .7
PAPi > 1.5
RA < 15
PCW < 15

Wean MCS

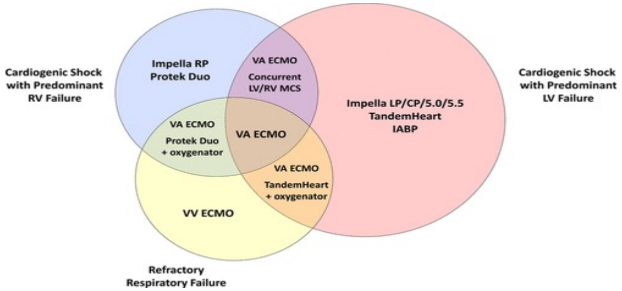

Ochsner Health System

Loma Linda University Medical Center

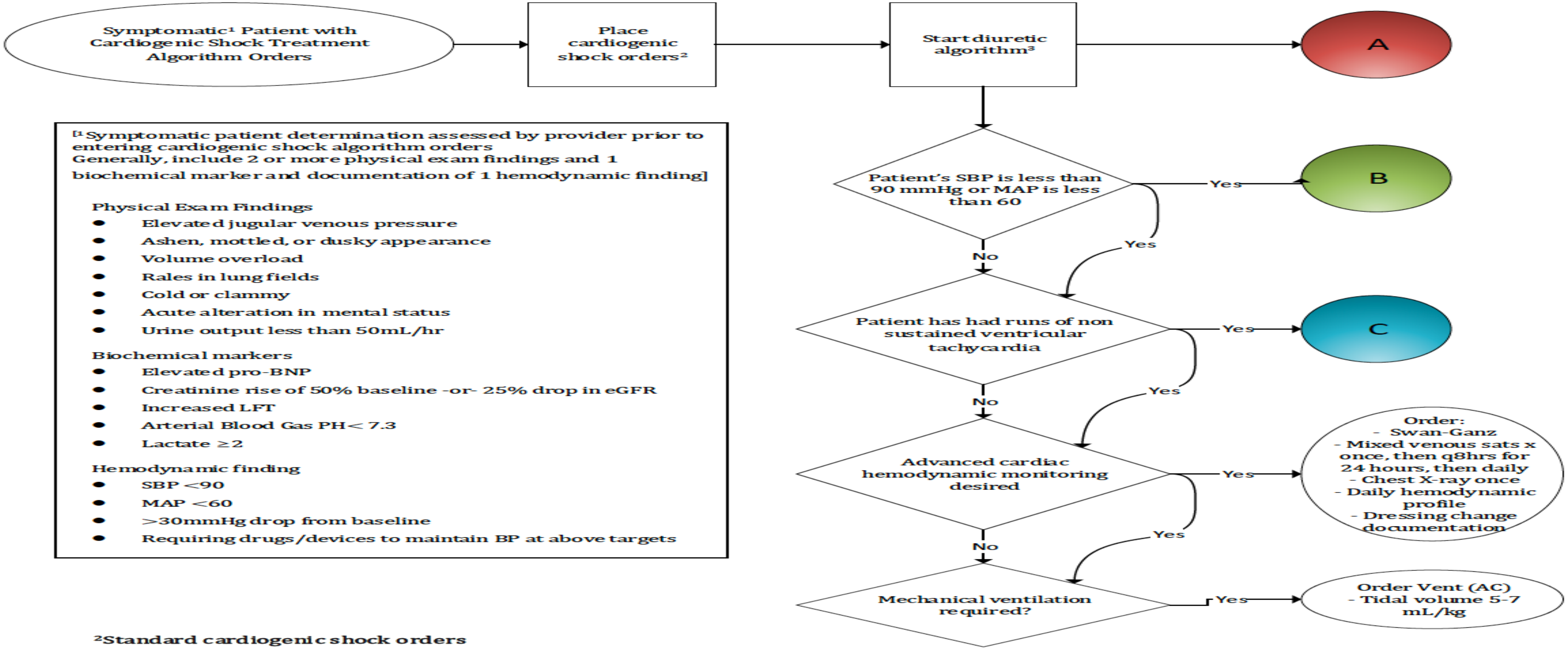

[[1] Symptomatic patient determination assessed by provider prior to entering cardiogenic shock algorithm orders
Generally, include 2 or more physical exam findings and 1 biochemical marker and documentation of 1 hemodynamic finding]

Physical Exam Findings

- Elevated jugular venous pressure
- Ashen, mottled, or dusky appearance
- Volume overload
- Rales in lung fields
- Cold or clammy
- Acute alteration in mental status
- Urine output less than 50mL/hr

Biochemical markers

- Elevated pro-BNP
- Creatinine rise of 50% baseline -or- 25% drop in eGFR
- Increased LFT
- Arterial Blood Gas PH < 7.3
- Lactate ≥ 2

Hemodynamic finding

- SBP <90
- MAP <60
- >30mmHg drop from baseline
- Requiring drugs/devices to maintain BP at above targets

[2] **Standard cardiogenic shock orders**

Add cardiogenic shock to active problem list<br>Order telemetry for 72 hours<br>Order Pulse Oximetry<br>Order measure weight daily<br>Order Intake and Output every 12 hours<br>Order Vital signs every hour<br>Order ABG once and every 8 hours for 24 hours then daily<br>Order Chest X-ray x once<br>Order [Complete Metabolic Panel, Complete Blood Cell count, PT/INR, Cardiac enzymes, Lactate] x Once<br>Order ECG x once<br>Order Echo x once<br>Order CMP daily<br>Order lactate Q8hr x3	**Add orders to notify cardiology fellow/attending:**<br>HR below 40 -or- above 150<br>MAP below 55 for 10 min<br>RR>20 for 3 min -or- O2 stat <90%<br>Average urine output below 50mL/hr for 4 hours<br>NSVT >10 beats<br>ABG PH<7.3<br>Lactate >5<br>Dobutamine >5 mcg/kg/min<br>Dopamine >5 mcg/kg/min<br>Starting norepinephrine<br>Starting epinephrine<br>Starting vasopressin

Baylor Scott and White Heart Hospital

Cardiogenic Shock Assessment Protocol

BaylorScott&White HEALTH

Objective: Early activation of the SHOCK team with consultation of the cardiology/advanced heart failure specialist to improve outcomes.

Workflow

1 Suspect cardiogenic shock
SALUDE
Exclusion criteria: DNAR and/or hospice care

↓

2 Complete cardiogenic shock workup, assess volume status and ensure secondary vascular access
ELCS

↓

3 **ACTIVATE SHOCK TEAM**

↓

4 Transfer to ICU (if not there) and place Swan Ganz

↓

5 **ASSESS**

a. Adjust inotropic and vasopressor support as necessary
b. Ensure advanced airway support for impending hypoxia and/or hypercapnia
c. Evaluate end organ perfusion
d. Determine need for higher level of care
e. Determine need for tMCS (see BSWH Cardiogenic Shock Mechanical Support Device Protocol)

*CV or HF fellow or attending depending upon site

Suspicion of cardiogenic shock

S<br>*SBP*	A<br>*Arrhythmias*	L<br>*Labs*	U<br>*Urine output/function*	D<br>*Drips*	E<br>*Examination*
☑ SBP <90 mmHg (known baseline SBP >100 mmHg)<br>**or**<br>☑ ↘ in SBP >20% for ≥30 minutes before intervention	☑ Frequent PVCs or sustained VT<br>☑ *Unexplained supraventricular tachycardia*	☑ Troponin<br>☑ ST elevation/ depression<br>☑ ↗Cr >0.5 mg/dl within 24 hrs (baseline Cr <2.5 mg/dl)	☑ Cr↗ >0.5 mg/dl within 24 hrs<br>☑ Oliguria/anuria (urine output) <400 mL/24 h or <17 mL/h	☑ Drips: Empiric use of vasopressors, vasodilators<br>**or**<br>☑ IABP	☑ Cool/ clammy skin<br>☑ Peripheral cyanosis<br>☑ Altered mental status<br>☑ Respiratory distress

Diagnosing of cardiogenic shock

E<br>*EKG & ECHO*	L<br>*Labs (added)*	C<br>*Consult*	→ S<br>*Swan Ganz*
• 12-lead EKG<br>• STAT ECHO	• Lactate >2<br>• pH <7.35<br>• Central venous blood gas<br>• Procalcitonin<br>*If not drawn already:*<br>• *Troponin with high PTP**<br>• *Metabolic panel*<br>• *LFTs*<br>• *CBC*<br>• *BNP (or pro BNP)*	• Review findings with cardiogenic shock MD*<br>• **Goal:** Confirm need for Swan Ganz	• Insert a Swan Ganz (if no Swan Ganz is available, draw a venous blood gas)<br>• CI <2.2 on Inotropes<br>• CI <2.0 without Inotropes<br>• PCWP >15<br>• CPO <0.6<br>• PAPi <0.9<br>• CVP/PCWP <0.6

Cardiac Power Output (CPO) = MAP x CO/451

Pulmonary Artery Pulsatility Index (PAPI) = (sPAP-dPAP)/RA

BSWH Cardiogenic Shock Collaborative

Cardiogenic Shock Mechanical Support Device Protocol

BaylorScott&White HEALTH

Objective: Provide prompt mechanical ventricular unloading management and appropriate device utilization to maximize outcomes.

Criteria for refractory shock

- Lactate >3
- Urine output <30cc/hr
- CPO <0.6
- Increasing pressor requirement
- Evidence of organ hypo-perfusion

Criteria for RV dysfunction

- PAPi <1.0
- RA >15mmHg
- RA/PCWP ratio >0.63

Common resuscitation medications

- Dopamine 2-10 mcg/kg/min
- Epi 0.02-0.08 mcg/kg/min
- Norepi 0.02-0.3 mcg/kg/min
- Milrinone 0.125-0.5 mcg/kg/min
- Dobutamine 2-7.5 mcg/kg/min
- Vasopressin 40 units IV

Vascular access assessment

- Evaluate history of PAD
- Obesity
- Groin access consideration
- R/O LV thrombus

Note: IABP should only be considered if no other support device is available.

*Consideration should be made regarding timing of implant, bridge to transplant, bridge to LVAD.

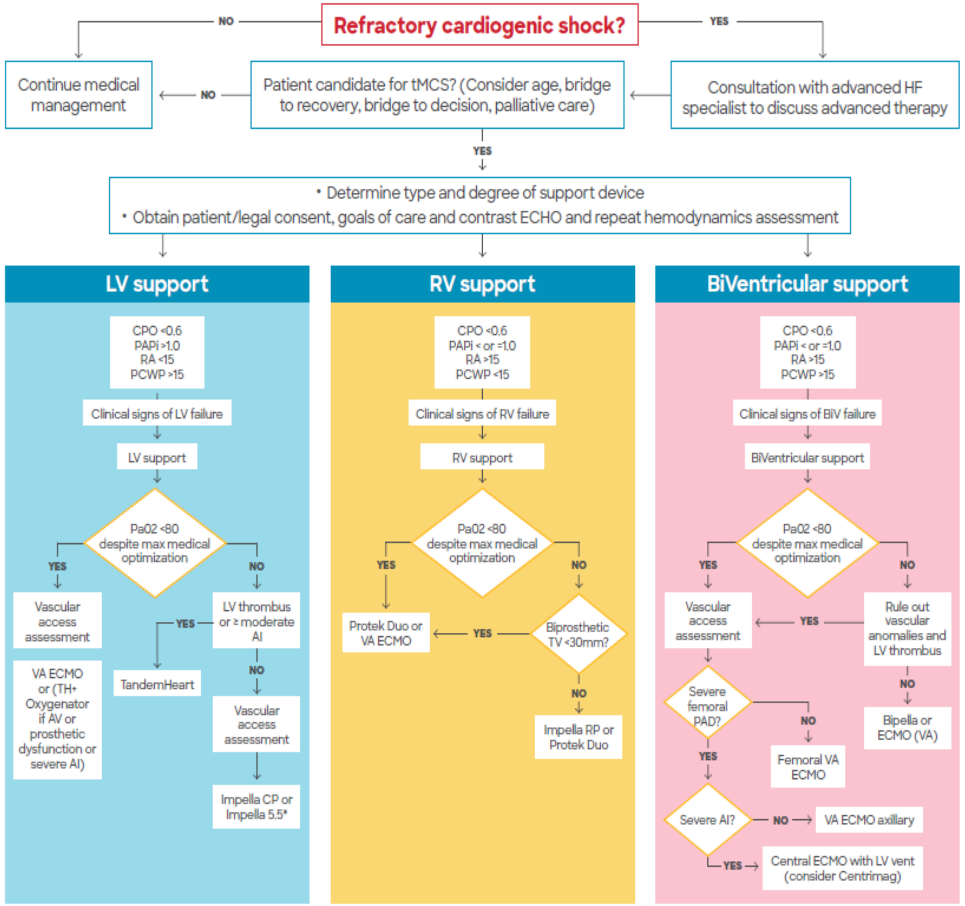

Cardiac Power Output (CPO) = MAP x CO/451

Pulmonary Artery Pulsatility Index (PAPI) = (sPAP-dPAP)/RA

BSWH Cardiogenic Shock Collaborative

Supplement: Interview Questions

- Demographics of Hospital (describe): Location of hospital. Number of beds/ICU/Training hospital or not VAD program/ Transplant program.
- Why did you start a CS program?
- If you had to teach someone to start a CS program right now? What would you tell them?
- What are the essential components that a CS program needs? (e.g. one call, shock personnel, protocols, quality review)
- How did you get buy in: administration, partners, other service lines.
- If you can go back in time, how would you do it differently?
- What educational methods were implemented to promote shock algorithms?
- Was there any industry help?
- How long did it take to be launch shock program? What do you think is a reasonable time frame for someone to launch a program?
- What were some early problems to starting? What were the solutions?
- How old is your CS program?
- Who is on the team?
- What happens during a shock call?
- 3 scenarios: What does shock activation look like from cath lab, ER and from outside hospitals?
- How many other hospitals do you partner with?
- Who performs pulmonary artery catheter insertion?

Supplement: Interview Questions

- What CS protocol are you using?
- What MCS is used? What escalation strategies are there?
- Post activation what does care look like it look like? (Pa catheter, labs, nursing intervention)
- Who manages the patient in the ICU? (ICU team, advanced CHF team)
- What was the hardest case you ever had?
- Is there a separate ECMO team?
- Is there Advanced HF specialist?
- Tell me about your review process.
- How do you stage patients for SCAI? – presentation to your hospital or outside
- What are the important parts of the review process?
- What metrics does your shock program use?
- How are shock cases identified, especially if there is no activation?
- Are you part of a registry? If so which one?
- What was it like during the first year of the program?
- Did your program grow the second year?
- How did you grow it? What was done for outreach?
- Do those other hospitals have protocols for transfer?
- What does transfer of shock patients look like? Is there a flight team, do you have your own flight team?
- For hospitals with Impella only what would you tell them? Such as partnering with your shock program?
